# Biotechnological Road Map for Innovative Weed Management

**DOI:** 10.3389/fpls.2022.887723

**Published:** 2022-04-25

**Authors:** Albert Chern Sun Wong, Karen Massel, Yasmine Lam, Jessica Hintzsche, Bhagirath Singh Chauhan

**Affiliations:** ^1^Centre for Crop Science, Queensland Alliance for Agriculture and Food Innovation, The University of Queensland, Brisbane, QLD, Australia; ^2^Centre for Crop Science, Queensland Alliance for Agriculture and Food Innovation, The University of Queensland, Gatton, QLD, Australia; ^3^School of Agriculture and Food Sciences, The University of Queensland, Gatton, QLD, Australia

**Keywords:** weed, CRISPR, genomics, biotechnology, herbicide, transient, RNAi, gene drive

## Abstract

In most agriculture farmlands, weed management is predominantly reliant on integrated weed management (IWM) strategies, such as herbicide application. However, the overuse and misuse of herbicides, coupled with the lack of novel active ingredients, has resulted in the uptrend of herbicide-resistant weeds globally. Moreover, weedy traits that contribute to weed seed bank persistence further exacerbate the challenges in weed management. Despite ongoing efforts in identifying and improving current weed management processes, the pressing need for novel control techniques in agricultural weed management should not be overlooked. The advent of CRISPR/Cas9 gene-editing systems, coupled with the recent advances in “omics” and cheaper sequencing technologies, has brought into focus the potential of managing weeds in farmlands through direct genetic control approaches, but could be achieved stably or transiently. These approaches encompass a range of technologies that could potentially manipulate expression of key genes in weeds to reduce its fitness and competitiveness, or, by altering the crop to improve its competitiveness or herbicide tolerance. The push for reducing or circumventing the use of chemicals in farmlands has provided an added incentive to develop practical and feasible molecular approaches for weed management, although there are significant technical, practical, and regulatory challenges for utilizing these prospective molecular technologies in weed management.

## Introduction

The world population is projected to increase from the current average of 7.7 billion people in 2018–2020 to 8.5 billion people in 2030 (OECD/FAO, 2021). Population growth is one of the key drivers affecting global agricultural commodities for food and non-food demand. One of the most significant challenges facing crop improvement programs globally is the capacity to adequately match crop production with demand, thereby ensuring food security. Global crop production is encumbered by various abiotic and biotic stresses which are further exacerbated by climate change. It is evident that innovative approaches and technologies are urgently needed to address these issues, ensuring global crop production can meet the expected world population increase in the coming years.

Numerous initiatives spearheaded by various research institutes, private companies, and philanthropic organizations to tackle both abiotic and biotic stresses are currently underway. Many of these initiatives involve the use of recent advances in genome editing to improve crop resilience and adaptability to various environments, improve yields in suboptimal conditions, and increase crops’ resistance to pathogens and insect pests. To date, several promising findings, such as the alterations of plant architecture, increased drought adaptation capabilities, increased salt tolerance, and increased pest and disease resistance have been reported (Wang et al., 2014; Yin and Qiu, 2019; Zhang et al., 2019; Tyagi et al., 2020; Zeng et al., 2020; Massel et al., 2021). A recent report on the transgenic expression of the human RNA demethylase *FTO* (fat mass and obesity associated) gene in rice and potato have resulted in an astonishingly ~50% increase in yield (Yu et al., 2021), although the approach involved would be considered as genetically modified (GM) and will fall under GM regulations.

Many molecular strategies for crop improvements have been largely focused on the improvement of crop resilience, adaptability, and yield, such as improving resistance to pathogens and insect pests. However, an equally pressing issue in farming is the control of weeds in agricultural lands. Weeds are a detrimental threat to global crop production in both developing and developed countries (Chauhan, 2020). Overall, among the biotic factors causing crop losses, weeds contribute to the highest potential yield loss to crops, followed by animal pests (insects, mites, nematodes, birds, rodents, etc.) and pathogens (fungi, viruses, bacteria, etc., Oerke, 2006). Annual crop losses and cost of weeds have been estimated to be at AUD 3.3 billion in Australia and USD 33 billion in the United States (Pimentel et al., 2005; Lewellyn et al., 2016).

Some molecular approaches have been implemented in conjunction with herbicide application to reduce the proliferation of weeds in agricultural lands. One such approach is the development of herbicide-resistant crops, such as the well-known Roundup Ready® crops (Padgette et al., 1995, 1996; Barry et al., 1997). The development of glyphosate-resistant crops enables the application of glyphosate, a non-selective herbicide, to eliminate unwanted weeds in the field at various application timings, thus enhancing the level of weed control. However, the emergence of herbicide-resistant GM crops has also contributed to the lack of novel herbicides discovery as it encourages the use of existing herbicides (Duke, 2012). Other factors such as the banning of currently used herbicides, high cost of new active ingredients discovery and marketing further discourage the development of novel herbicides (Duke, 2012; Székács, 2021). Nevertheless, ongoing research for novel mode-of-action herbicides is crucial as it provides alternative tools to combat and circumvent current trends of herbicide-resistant weeds. For example, in the lysine biosynthesis pathway which remains largely unexplored for herbicide development, novel inhibitors that target dihydrodipicolinate synthase (DHDPS), which catalyzes the first and rate-limiting step in lysine biosynthesis has been reported (Da Costa et al., 2021).

Gene discovery, “omics,” and genome editing technologies currently applied in crop research can be potentially applied to weeds as tools for weed management. However, unlike in crop improvement, the utilization of molecular technologies to control weeds poses many challenges. These challenges include concerns surrounding the use and regulation of GM technologies in managing weeds and non-crop plant species in the wild, and the potential ecological risks posed by the intentional release of GM plant materials (Neve, 2018; Westwood et al., 2018; Barrett et al., 2019). Aside from GM methods, transient technologies relying on the non-transformative applications of RNA interference (RNAi) mechanism are also potential molecular approaches to control weeds instead of heavy reliance on herbicides. To date, significant advances in this technology have been made in crop pest and disease management (Cagliari et al., 2019).

The ongoing challenges in controlling weed-related damage to agriculture production have highlighted the need for new avenues to control weeds, other than relying on the conventional use of herbicides. Weed control technology must continuously improve to stay ahead of weed adaptation and evolution, and molecular approaches could potentially be explored as tools to control weeds. This review discusses the current challenges in managing herbicide resistance in weeds, and the molecular approaches that could be integrated into current strategies and aid in future weed management. Molecular approaches, including CRISPR/Cas9, gene drives and RNAi technology, are discussed in this review, along with a proposed list of potential gene targets for future molecular research on weed management.

## Challenges in Weed Management

Weed management is challenging due to the diversity of weed and crop species and the various agricultural climates that these crops can be sown. There is no “one size fits all” model for any cropping system. The application of mechanical or chemical control methods alone has failed to lead to a sufficient suppression of weeds. However, integrated weed management (IWM) approaches, which encompasses coordinated application of various mechanical, chemical, and biological control methods, can help reduce weed seed bank and provide environmental and economic benefits in the long run (Harker and O’Donovan, 2013; Knezevic et al., 2017; Jabran and Chauhan, 2018; Alagbo et al., 2022). Despite the usefulness of IWM, such strategies need to be heavily researched to determine the appropriate cultural, physical, and chemical methods that would be the most beneficial for the agroecological zone. Additionally, the change in the global climate has rendered some tried and true practices ineffective, leaving the door open to innovation in IWM.

Climate change has raised complications in a number of different agricultural systems, and many of the challenges with weed management will be intensified which have been summarized in Ramesh et al. (2017). Firstly, with the expected reduction in rainfall in already dry regions, the resilience of crops will be encumbered. In this scenario, weeds have mechanisms to allow them to combat such stressors and out-compete the struggling crops, while also having extended periods of growth beyond their usual growing season (Peters and Gerowitt, 2014; Ramesh et al., 2017). This is also partially linked to their ability to quickly accumulate mutations to be better adapted to rapidly changing climate scenarios, in contrast to many crops which rely on breeding programs to introgress desired traits in a relatively slow manner. Focusing more on the management side, climate change is expected to result in the need for new weed management strategies that will need to be rapidly implemented to be an effective combatant to the rapid climate variance. The change in climate will also result in the increased instability of current herbicides. For example, suddenly warmer regions will need to implement herbicides with higher heat tolerances or spray strategies will need to be altered to navigate new patterns of rainfall. Thus, from the examples highlighted above, the potential to further agitate weed management systems due to climate change can be seen. In addition to the compounding effects of climate change on weed management, the following review highlights some of the traits that allow for weeds to be so hardy, in addition to the already known complications prominent within weed management.

### Herbicide Resistance in Weeds

The increased occurrence of various herbicide-resistant weeds in agricultural lands is one of the major issues faced in weed management. Human interventions and farming practices, such as the massive adoption of herbicide-based technologies to control weeds over large farmlands, have contributed to the evolution of herbicide resistance in many weeds (Cardina et al., 2002; Roux and Reboud, 2007). This is especially the case with continuous and non-judicious use of herbicides with the same mode of action. Biological factors that include the genetics, life cycle, and ecology of weeds also play a part in the evolution of their herbicide resistance mechanisms. Furthermore, no new mode of action herbicide has been released in the market for more than 30 years (Duke, 2012), which adds further pressure in controlling the increased number of herbicide-resistant weeds globally. Although herbicides with new modes of action, such as cinmethylin (Campe et al., 2018), tetflupyrolimet (Dayan, 2019; Dayan et al., 2019), and cyclopyrimorate (Shino et al., 2021) have been developed, weed control cannot be heavily dependent on utilizing novel herbicides as weeds can also develop resistance. From 1957 to 2020, the global reported number of unique cases of herbicide-resistant weeds has increased from 2 to 507 (Heap, 2022). In general, herbicide resistance mechanisms can be categorized into two broad types: (1) target-site resistance, and (2) non-target site resistance.

Target-site resistance typically involves specific site mutations in the target enzyme, which prevents herbicide from binding to the target enzyme. Mutations could occur in the binding sites within the enzyme, or on other parts of the enzyme which could alter the conformation of the enzyme in ways that the herbicide could no longer inhibit the activity of the enzyme. Other forms of target-site resistance include target gene amplification (the increase in target gene copies) and the increase in target gene expression. These resistance mechanisms aim to increase the production capacity and abundance of the target enzyme, in which higher doses of a herbicide would be required to fully inhibit the target enzyme. For example, gene amplification of the herbicide target gene 5-enolpyruvylshikimate-3-phosphate synthase (EPSPS) has been reported in weed species, such as *Amaranthus palmeri* (Gaines et al., 2010), *Chloris truncata* (Ngo et al., 2018), and *Hordeum glaucum* (Adu-Yeboah et al., 2020), whereas ACCase gene amplification has been reported in *Digitaria sanguinalis* (Laforest et al., 2017).

Non-target site resistance stems from the physiological characteristics of the plant and how it absorbs, metabolizes, and/or sequesters the herbicide (Jugulam and Shyam, 2019). As opposed to target-site resistance mechanisms, non-target site resistance is significantly more challenging to identify, as reducing the concentration of the herbicides entering and remaining in the plant systems usually involve multiple gene families controlling key processes such as metabolism, translocation, and sequestration of the herbicide molecules. Cases of weeds that have evolved non-target site resistance against major herbicide groups have been summarized in recent literature (Gaines et al., 2020; Perotti et al., 2020). For example, enhanced metabolism of the herbicide molecules is associated with the proteins, such as cytochrome P-450 monooxygenases (P450s), glutathione-S-transferases (GSTs) and/or glycosyl-transferases (GTs), which are involved in the various phases of herbicide detoxification (Gaines et al., 2020; Perotti et al., 2020). P450s form one of the largest gene families in plants and are vital to plant development in defense, having roles in the synthesis of hormones, lipids, primary and secondary metabolites, and metabolisms of various compounds (Mizutani, 2012; Xu et al., 2015). However, in terms of herbicide metabolism, they participate in the first phase by modifying the chemical functional groups of the herbicide molecules, enabling the conjugation of the herbicide molecules *via* GSTs or GTs to thiols groups or glucose (Cummins et al., 2013; Chronopoulou et al., 2017). Conjugated herbicide molecules are then transported to vacuoles *via* transporter proteins, such as the ATP-binding cassette (ABC) proteins (Martinoia et al., 1993; Theodoulou, 2000; Conte and Lloyd, 2011), and cation amino acid transporter (CAT; Su et al., 2004; Jóri et al., 2007), for compartmentalization and degradation.

Another example of non-target site resistance is through reducing translocation of the herbicide, so once the herbicide enters the source leaves they are prevented from reaching the growing and meristematic tissues *via* the phloem and/or xylem. Reduced translocation can be due to sequestration, which traps the herbicide molecules within the source tissues, or altered activity of transporter proteins, which either prevent or limit the entrance of the herbicide molecules into the phloem and/or xylem (Délye, 2013). Reduced translocation of glyphosate (Ge et al., 2011, 2012; Moretti and Hanson, 2017), paraquat (Yu et al., 2004, 2010; Brunharo and Hanson, 2017; Moretti and Hanson, 2017), and 2,4-D (Riar et al., 2011; Goggin et al., 2016) have been reported in different weed species.

### Weed Seed Bank Persistence

Most weed species are known to be hardy and persistent in nature, producing thousands of seeds that can withstand various adverse environmental conditions, while staying dormant in the soil for long periods (Manalil and Chauhan, 2021; Chauhan and Manalil, 2022). When optimal germination conditions are met, the seeds will germinate and compete with the crops sown on the same area of land. This makes weed management challenging, such as the application of selective herbicides when both the weeds and the crop in the farmland belong to the same group of flowering plants (e.g., monocot weed species growing within cereal crops). Consistent application of the range of control methods in IWM can be a long-term solution for minimizing weed seed bank, although only a limited number of studies on weed seed bank corresponding to management are available (Sosnoskie et al., 2009; Schwartz et al., 2015). Additionally, in agricultural farmlands, weed seed bank can contain seeds of multiple different weed species. Every weed species has their own biology, life cycle, and ecology, which in turn would require different IWM approaches.

Seed dormancy is the main contributor to a persistent weed seed bank globally and is a trait with high plasticity in weed species, thus, making weed control difficult to achieve as it adjusts the weed population to a cropping system (Baskin and Baskin, 2006; Schwartz-Lazaro and Copes, 2019). Dormancy can be categorized into two types: (1) primary dormancy, where dormancy is induced during seed development and prior to dispersion from the mother plant, and (2) secondary dormancy, when the dispersed seeds are met with suboptimal environmental conditions for germination (Carmona, 1992; Vivian et al., 2008). While seed dormancy is also a heritable genetic trait, it is complex to study due to the trait’s genetic and environmental (G × E) interactions (Foley and Fennimore, 1998). Nevertheless, recent genetic and molecular studies on seed dormancy using model plant species have provided important genomic information to aid the understanding of seed dormancy in weeds (Gu et al., 2018; Pipatpongpinyo et al., 2020), and genes that are involved in the regulation of seed dormancy have been extensively reviewed (Graeber et al., 2012; Nonogaki, 2014; Klupczyńska and Pawłowski, 2021).

### Lack of Genomic Resources in Weeds

Major obstacles in implementing molecular approaches for weed management include the lack of genomic resources on many major weeds, which encompasses the lack of genomic and molecular studies on weeds relative to many crop plants. Tools from genomics and molecular biology should be utilized to obtain genomic information on weeds, which can aid in the investigation of herbicide resistance mechanisms.

Initiatives such as the International Weed Genomics Consortium^1^ have begun to fully sequence several major weed species in recent years, such as *Lolium rigidum* and *Conyza sumatrensis* (Manning, 2021). This initiative and future works in addressing greater availability of genomic resources of various major weed species would be beneficial not only for the development of molecular approaches for diagnostic and weed management, but also for a better understanding of weed biology, weedy traits, and the adaptive evolution of herbicide resistance (Ravet et al., 2018). Genomic resources of these major weed species could also be important in revealing potential genetic resources that could be utilized for future crop breeding for the integration of beneficial traits from weed species into crops. Harnessing the genetic information of weed species also enables a better understanding of weeds’ biotic and abiotic tolerance. As most weeds are extremely tolerant to various harsh environments, generating and studying the genomic resources of these weeds could also aid in the understanding of stress tolerance and possibly be applied to related crops. Other genomic information such as population genetics can also potentially contribute to management decisions, such as the choice of herbicides and herbicide rotation (Perotti et al., 2020).

## Potential Molecular Approaches Targeting Weeds to Control Fitness

**Figure 1:**
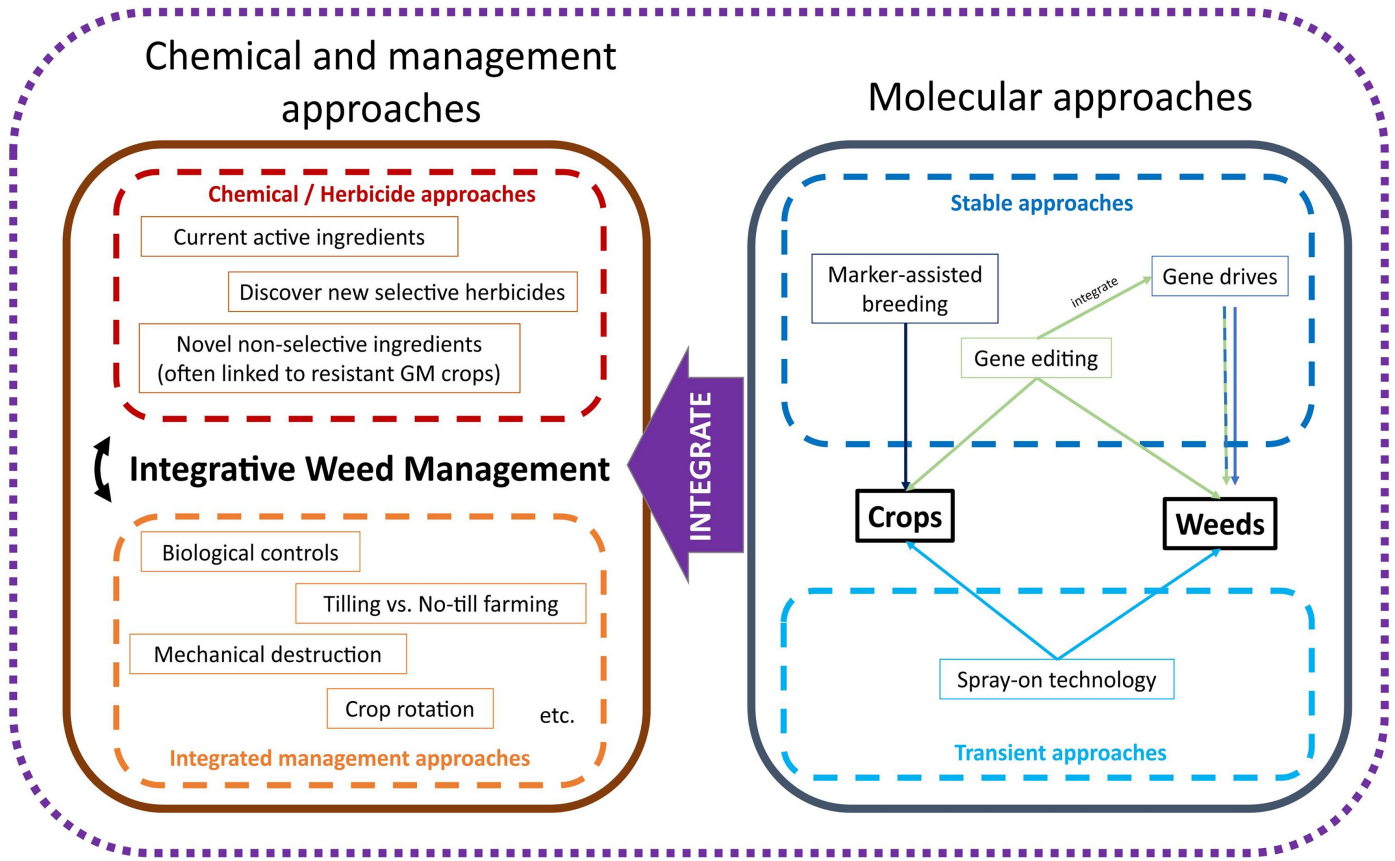
A conceptual framework for innovative weed management. Conventional approaches using chemicals and farm management are often applied together and can be improved or integrated with biotechnological approaches for future weed management. Biotechnological weed management can be applied to crops or weeds *via* stable and transient approaches.

In the current context of agriculture, the goal of studying weed biology and physiology is to understand the habitat, life cycle, propagation, and proliferation patterns of weeds, while applying these to reduce their fitness and colonization in agricultural farmlands. Herbicide application and other management-based approaches are means to reduce the fitness of the weed population in agricultural lands. Long-term objectives of weed management would be to reduce the global weed seed banks, effectively controlling the weed populations rather than treating the “symptom” of managing weeds as they appear. Numerous genetic approaches could be implemented which could be used to improve weed management in the future (Figure 1). These strategies combine genomics and biotechnological tools and could be implemented in either the crop or weed species depending on the desired outcome.

### Genomics Tools

The increasing level of “omics” data available in weed species enables researchers to gain better insights into weedy characteristics such as dormancy, invasiveness, and herbicide tolerance/susceptibility mechanisms. In the case of glyphosate resistance in *Ipomoea purpurea*, using targeted exome re-sequencing, Van Etten et al. (2020) found no mutations in the expected glyphosate target protein of EPSPS, but instead found selective sweeps in other genes involved in herbicide detoxification which varied by population. Therefore, although the physiological mechanism is the same for glyphosate resistance in this weed, varied populations of divergent mechanisms for herbicide detoxification are present. Understanding the evolved herbicide resistance tactics of weeds can provide prospective genes and gene networks that could be manipulated in a diverse range of crops for alternative herbicide tolerance mechanisms.

Advances in genomics tools and resources for weeds will be crucial for the development of various molecular approaches for weed management. Cumulative efforts in building genomic resources for weeds will form the basis for the development of plant transformation and gene editing protocols for gene function studies. It can also help scientists to better understand complex traits such as abiotic stresses, and various non-target site resistance mechanisms employed by weeds (Ravet et al., 2018). However, the development of genomic tools and “omics” database for weeds poses several challenges. These include the complexities in establishing the understanding of underlying biology across the large diversity of weed species and the diverse nature of weed-living systems (Martin et al., 2019; Patterson et al., 2019). Furthermore, unlike reference model plant species and other well-studied crop species, obtaining the desired genotypes (e.g., highly homogenous lines) for the development of genomic reference materials is encumbered by the maintenance of large genetic diversity in weeds (Basu et al., 2004; Stewart et al., 2009; Vigueira et al., 2013).

Marker-assisted crop breeding has led to enormous genetic gains for numerous traits such as disease resistance and yields, and have the potential to be applied for weed management strategies. Researchers can use Genome-Wide Association Studies (GWAS) to associate mutations in genetic elements to key traits for improved weed management, such as herbicide tolerance or those that allow crops to outcompete weeds. This strategy has been successfully applied to crop species such as wheat (Shi et al., 2020; Xu et al., 2020), cotton (Thyssen et al., 2014, 2018), sorghum (Adhikari et al., 2020), and fababean (Abou-Khater et al., 2022) for varied natural herbicide tolerance.

Similarly, novel genetic variation can be induced using chemical mutagenesis to create mutant populations, in turn producing new allelic variants and/or discovering new modes of action for weed control. Implementation of this method can be seen where imidazolidinone tolerance in wheat (Newhouse et al., 1992) and chickpea (Croser et al., 2021) was produced using ethyl methanesulfonate (EMS) mutant population. *Leucaena leucocephala* is another example of the implementation of chemical mutagenesis for weed management. Normally a pasture crop in Northern Australia, *Leucaena leucocephala* is known to be a rampant weed in other regions. In an attempt to combat this weed, a mutagenized population was created to develop various sterile alternatives, including cytoplasmic male sterile and a triploid variety (Mcmillan et al., 2019). Although EMS mutagenesis can achieve a non-GM outcome in an elite crop variety and provide a rapid strategy to market, the effects of this approach on agronomic/quality traits will need further evaluation.

### CRISPR/Cas9

The CRISPR/Cas9 approach has been one of the most used technologies for genetic modification of crops and other organisms. It is a flexible and versatile option for highly targeted modification of genomes, mostly applied to disrupt gene function (Zhang et al., 2018). First, it creates highly targeted Double-Stranded Breaks (DSB) within the host genome with the introduction of two CRISPR components: a guide RNA (gRNA) and a CRISPR Associated Nuclease (Cas). The Cas protein contains two nuclease domains which each cut one strand of the DNA, targeted to the specific location in the genome through the small non-coding gRNA. Targeting potential of the gRNA limited to regions directly upstream of a Cas-dependent Protospacer Adjacent Motif (PAM), which for the most common Cas9 from *Streptococcus pyogenes* is 5′-NGG-3′. Together, these components facilitate targeted DSBs, forcing the plant to repair the break by Non-Homologous End Joining (NHEJ) or Homology-Directed Repair (HDR). The first and most predominant in plants, NHEJ, fixes the break by ligating the broken ends together, but often does so erroneously. If targeted to the coding region of a gene, a frameshift mutation will lead to a gene knockout. The randomness of NHEJ repair means the outcomes are often viewed synonymously with mutagenesis or natural mutation, allowing gene-edited products to avoid the regulatory constraints of being classified as genetically modified.

The application of CRISPR/Cas9 in crop improvement has been extensively reviewed (Chen et al., 2019; Massel et al., 2021). Modified versions of the Cas9 proteins have also resulted in newer technologies such as base editing and prime editing for precise genome editing (Molla et al., 2021). The CRISPR/Cas9 approach is certainly a promising tool that could be utilized and adapted for weed management in two ways. The first would be to modify the genomes of the crop to boost its performance and outcompete weeds, through mechanisms such as increased herbicide tolerance, improved early vigor, or through allelopathic means to reduce weed establishment. Alternatively, one could implement gene editing strategies to the weed itself to alter its development or herbicide tolerance.

The delivery of the CRISPR/Cas9 constructs often requires the establishment of efficient transformation systems for many of these major weed species. Plant transformation systems are expensive to develop, time-consuming, and often genotype-specific. Developing plant transformation systems for the appropriate weeds would be highly challenging. In addition to optimizing plant transformation systems for these weed species, the delivery of the CRISPR/Cas9 constructs editing targeted genes in specific weed species, and the propagation of weed species carrying these constructs will be difficult from a regulatory standpoint. Although GM regulation could be avoided if the CRISPR/Cas9 editing components could be segregated out from the transgenic population prior to releasing into the wild for propagation, the regulations are highly complex and vary globally. Nevertheless, the rapid advancement of CRISPR/Cas9 technology could be useful for designing synthetic gene drives that could potentially be used for population and fitness control in weeds.

### Gene Drive

Gene drive refers to the process which sequences of DNA are biasedly inherited in their favor and circumventing Mendelians inheritance, which results in a preferential increase of a specific genotype (Burt and Trivers, 2006; National Academies of Sciences, Engineering, and Medicine, 2016; Alphey et al., 2020). They are able to spread through populations, even when they impose a fitness cost on their host (Lindholm et al., 2016). Gene drives exist in nature through a variety of mechanisms, such as meiotic drives, transposable elements, and homing endonuclease genes (HEGs). HEGs were first suggested as tools that can be used for generating synthetic gene drives (Burt, 2003), and were first used in gene-drive systems in strains of *Drosophila* (Rong and Golic, 2003; Chan et al., 2013) or anopheline mosquitoes (Windbichler et al., 2011).

The idea of using gene drives for weed management is not new, as it has been discussed in several studies (Neve, 2018; Westwood et al., 2018; Barrett et al., 2019). Unlike the implementation of gene drives in controlling insect populations, the utilization of gene drives to control weeds faces significant challenges caused by the diversity of weed biology, and technical difficulties in developing efficient gene drive that can work in plants. Gene drives enable the spread of specific alleles only over generations; thus, the utilization of gene drives will be most effective in organisms that can reproduce quickly or that are highly dispersed. Unfortunately, not all weed species can be selected for genetic management *via* gene drive, due to different life-history factors. This includes modes of reproduction (sexual, asexual, or hermaphrodite), modes of crossing (inbreeding or outcrossing), modes of seed/pollen dispersal, seed dormancy, and genetic architecture such as polyploidy.

An efficient gene drive would require good cutting specificities so that the intended genetic change can be accurately inherited onto the progeny. The subsequent discovery of targeted genome editing tools, such as CRISPR/Cas9, have further improved cutting specificities and efficiencies of gene drive systems. CRISPR/Cas9-based gene drives have been successfully demonstrated in bacteria (Valderrama et al., 2019), yeast (Dicarlo et al., 2015), insects (Gantz and Bier, 2015; Gantz et al., 2015; Hammond et al., 2016; Kyrou et al., 2018), mice (Grunwald et al., 2019), and most recently in *Arabidopsis* (Zhang et al., 2021). Furthermore, a successful CRISPR/Cas9-based gene drive requires an efficient HDR pathway, instead of the NHEJ pathway (Gantz and Bier, 2015). In plants, HDR can be difficult to achieve as NHEJ is predominantly used to repair double-strand breaks (Puchta, 2004; Huang and Puchta, 2019). However, it has been shown that factors such as the amount of donor template, the concentration of Cas9 protein in the cell, and the timing of generating double-stranded breaks determine HDR efficiency in plants (Čermák et al., 2015; Gil-Humanes et al., 2017; Miki et al., 2018; Chen et al., 2019; Peng et al., 2020). The regulatory context will determine how this technology is able to be deployed in different jurisdictions and the extent to which societies (and markets) accept the use of gene drives, as HDR gene editing is often viewed as a GM outcome and subject to strict regulations.

By overcoming the biology and technical challenges of developing gene drive systems for weed management, gene drives can be used to (1) suppress the weed population, or (2) sensitize the weed population. Suppressing mechanisms refer to proliferating the mutation of crucial genes that will reduce the fitness of the weed population, thereby reducing their capability to compete with crops. The sensitizing approach refers to specifically reintroducing the herbicide susceptibility back into the resistant weed population.

### Transient Technology

Transient technology allows the user to temporarily manipulate the gene expression of the plant without making any stable changes to the genomic DNA. This means that outcomes are not subjected to strict GM regulations, significantly less development time is required, and do not rely on having tissue culture systems in place to modify the genome. Further, these systems can often be applied to weeds or crops that are already growing to modify traits on-the-spot, rather than requiring a gene drive system to spread the desired changes throughout the population.

The utilization of a double-stranded RNA (dsRNA) spray packaging has been shown to improve a range of management options for diseases and insects across a range of plant species (Mitter et al., 2017). This approach packages a dsRNA capable of targeting and downregulating the expression of key genes in the host plant involved in disease/pest growth. There is enormous potential for a similar approach to be applied to control weeds. One option is to use RNAi to target herbicide-resistant weeds, reducing the expression of their tolerance mechanism so previously developed herbicides will continue to work. Alternatively, this system could be adapted to target key genes that are solely found within weed species without impacting their expression in crops, whereby reducing the gene expression may reduce the competitiveness of the weed (e.g., development issues, loss of flowering, and reduced seed set).

Spray-on technologies have been rapidly advancing, where systems have been developed for transient expression of genes (or RNAi machinery) through packaging into viral vectors. Torti et al. (2021) demonstrated that target genes controlling growth and other physiological changes can be modified *via* the RNAi approach, and this may be applied to weed management. It is possible that a spray-on strategy could be used to specifically target either the crop or weed species using promoters that would only drive expression of genes in either plant. In terms of herbicide efficiency, one could imagine a scenario where a farmer could boost herbicide tolerance in the crop species without creating a stable genetic alteration that would not express in the weed species. Similarly, the weeds could be specifically targeted with an RNAi vector to silence key endogenous genes which would complement or replace the use of herbicides. Furthermore, the genes are non-transmissible to the next generation so different herbicide treatments could be applied over the years to reduce the emergence of herbicide-resistant weed populations (Mitter et al., 2017; Cagliari et al., 2019). Transient silencing/overexpression approaches are not expected to be regulated as GM products, thus they could be developed and released to potentially complement or replace current weed management strategies.

## Potential Gene Targets for Reducing Weed Fitness

Regardless of the challenges in implementing molecular approaches in weed management, either *via* genome editing approaches, or the dsRNA spray for transient editing, numerous prospective genes could be targeted for knockout and knockdown in weed species to reduce fitness, or conversely, genes targeted in the crop to improve fitness. Much of the challenge for spray-on technologies will be to ensure the transient alterations to gene expression are solely found in either the weed or crop species. As for the utilization of gene drives to release into the cropping environment, it will rely heavily on the successful creation of transgenic weeds carrying the gene drive, and the capability to drive the intended mutation into the weed population across several generations.

### Cytochrome P450 Family and Herbicide Target Genes

There is a wide range of potential gene targets that could be targeted by the abovementioned molecular approaches. Genes that will lead to various downstream phenotypic effects that reduce the plant’s survival and fitness when perturbed are often desirable targets for these approaches. For example, to improve herbicide efficiencies, one could consider altering genes within the cytochrome P450 family. These proteins have been shown to be upregulated in response to herbicide application (Pasquer et al., 2006; Hirose et al., 2007; Lu et al., 2015), which antagonizes the application of other herbicides, such as ACCases and acetolactate synthase (ALS) inhibitors (Peterson et al., 2016). Therefore, reducing the expression of P450s could potentially make the plant more susceptible to the herbicide, if the knockdown/out of this gene was not lethal.

However, although gene members of the cytochrome P450 family and other gene families (such as glutathione-S-transferases and glycosyl-transferases) involved in non-target site resistance are obvious targets for genetic manipulation, these gene families are often large and diverse (Martin et al., 2019), which makes targeting these genes specifically almost impractical. Thus, genetic resources from “omics” studies could also aid in revealing conditions and genetic elements that could be involved in the regulatory network of these superfamilies conferring herbicide resistance. For example, further understanding of how P450s are induced by herbicide application can be useful in designing vectors to exploit this mechanism. Hirose et al. (2007) has reported that the promoter of CYP72A21 in rice is activated when 2,4-D was applied, leading to increased CYP72A21 expression. One could consider altering the promoter region *via* gene editing to deactivate or suppress its sensitivity to 2,4-D application, which may avoid any constraints if a complete knockout is lethal. Thus, through targeting genes such as P450s, herbicide efficiency could be boosted by increasing the susceptibility in weed species.

### Plant Growth Regulator Genes

Two options for improving weed management are to reduce the competitiveness of the weed species or to improve the early vigor of the crop plant. Therefore, genes that are involved in primary functions of growth and development could be targeted for either trait. In terms of reducing weeds’ fitness and competitiveness, knockdown of these genes in weeds would be particularly useful in increasing their susceptibility to various biotic and abiotic stresses. For example, members of the phenylalanine ammonia-lyase (PAL) gene family could be targeted in the weed species. These genes are involved in the first step of the phenylpropanoid biosynthesis pathway, which leads to the synthesis of precursors of various primary and secondary metabolites important for growth and responses to various biotic and abiotic stresses, such as UV radiation, temperature, and pathogen infection (Edwards et al., 1985; Dixon and Paiva, 1995; Huang et al., 2010; Vogt, 2010; Kim and Hwang, 2014; Feng et al., 2022). Additionally, molecular components (e.g., transcription factors, hormone receptors, and transporters) that interact with plant hormones to regulate plant development could be selected as potential targets for reducing weed competitiveness. However, developing molecular tools targeting these components can be highly challenging due to the complex network of interactions between the molecular components and plant hormones (Domagalska and Leyser, 2011; Vanstraelen and Benková, 2012; Gallavotti, 2013; Schaller et al., 2015; Waldie and Leyser, 2018). Provided that challenges on developing genomic tools and resources for weeds can be overcome, the testing of gene drive systems and spray-on transient technologies targeting plant growth regulator genes in weeds could potentially complement many current weed management strategies.

Alternatively, improving early vigor of the crop plant may lead to improved growth which in turn, leads to suppression of weed growth (De Vida et al., 2006). Although in some instances, researchers have been searching for plants that can maintain high yields despite weed competition, this strategy further contributes to the ongoing weed seed bank issues. Therefore, weed-suppressive strategies and control methods employed in IWM are preferred. Additionally, there have been a few studies searching for QTLs for weed competitive traits in crops (Coleman et al., 2001; Bharamappanavara et al., 2020; Dimaano et al., 2020). Although many of these studies have not mentioned specific genes from fine mapping, it is likely that genes involved in growth and development like maturity genes, tillering genes, and leaf development genes will be strong candidates for improvement.

### Sex Determining and Flowering Time Genes

Plants possess diverse sexual systems that include obligate selfing, outcrossing, and apomixis. Different sexual systems are determined by their underlying genetics of temporal and spatial development of reproductive systems, resulting in sexual systems such as hermaphroditism, dioecism, monoecism and so on (Bawa and Beach, 1981; Charlesworth, 2002). Studies of the genetic basis of sex determining genes influencing floral and reproductive organs development in the genus *Silene* (Monéger, 2007; Bernasconi et al., 2009; Charlesworth, 2013), and the weed species of *Amaranthus tuberculatus* and *Amaranthus palmeri* (Montgomery et al., 2019, 2021), have provided gene targets that could potentially be tested for gene drive development and spray-on transient technology. Perturbing sex determining genes, including male-sterility and female-sterility factors, could potentially create an imbalance of sexes within the weed population, thus potentially causing the weed population to collapse. Interestingly, a flowering time gene, *FLOWERING LOCUS T* (*FT*) homolog is reported to be in the male-specific Y (MSY) region in the dioecious weed species of *Amaranthus tuberculatus* and *Amaranthus palmeri* (Montgomery et al., 2021), suggesting that perturbing the expression of this *FT* homolog could potentially alter the flowering time and affect fitness.

Flowering time genes have been extensively characterized in various plants, and the perturbation of these genes, such as *CONSTANS* (*CO*) and *FT*, result in abnormal timing of flowering and floral development (Koornneef et al., 1991; Putterill et al., 1995; Araki et al., 1998; Kobayashi et al., 1999; Kim et al., 2013). Disrupting the genetic sequence and expression of *CO* and *FT* homologs in weeds using gene drive and transient technology could potentially generate offspring with abnormal flowering time and floral development. This could directly affect the competitiveness of weeds in farmlands. However, it is important to note that the homologs of *CO*, *FT*, and their counterparts with similar amino acid sequences such as *CO*-like and *FT*-like genes in different plant species can possess various levels of functional redundancy (Yano et al., 2000; Izawa et al., 2002; Hayama and Coupland, 2004; Yoo et al., 2004; Hanzawa et al., 2005; Faure et al., 2007; Wong et al., 2014; Wolabu et al., 2016). Therefore, in-depth functional characterization of these genes in various weeds species would be required to test the feasibility of selecting flowering time genes as targets for molecular weed management.

### Seed Dormancy

Targeting seed dormancy genes is another obvious choice to inhibit seed fitness. In plants, there are varied combinations of seed dormancy genes that coordinate the control and longevity (Li and Foley, 1997; Nonogaki, 2014; Pipatpongpinyo et al., 2020). Oftentimes, these genes include transcription factors that induce flavonoid biosynthesis, production and accumulation of ABA, and gibberellic acid biosynthesis (Finch-Savage and Leubner-Metzger, 2006; Debeaujon et al., 2007). By implementing CRISPR/Cas9 targeting seed dormancy genes and incorporating into gene drives, it could potentially reduce the capability of the seed to germinate in the environment. This would work similarly to the “terminator technology” or genetic use restriction technology (GURT) to maintain the intellectual property of genetically modified materials (Visser et al., 2001; Lombardo, 2014). In this technology, there is a genetic switch that once released would mean the next generation of seeds are non-viable.

### Seed Shattering

Seed shattering is a key weedy trait that differentiates domesticated and wild plants (Dong and Wang, 2015). In crops, the retainment of inflorescence/pods is a staple of domestication which allows farmers to harvest the crops rather than the natural shedding of mature grains from the crops. The loss of the seed shattering trait would be important to reduce the spread of weeds across agricultural lands. A common mechanism in seed shattering in both monocots and dicots is the formation of the abscission layer in the inflorescence/pods *via* cell wall thickening and lignification (Harlan and deWet, 1965; Elgersma et al., 1988; Fuller and Allaby, 2009; Seymour et al., 2013; Dong and Wang, 2015). This mechanism and subsequent physiological processes leading to seed shattering are controlled by a complex network of plant signaling components involving plant hormones (Vivian-Smith and Koltunow, 1999). On the gene level, it has been reported that the loss-of-function mutation in the major seed shattering gene in sorghum (*Sh1*) and its ortholog in rice was selected for their non-shattering phenotypes (Lin et al., 2012; Lv et al., 2018; Li et al., 2019). Konishi et al. (2006) has reported that the non-shattering trait in domesticated rice can be caused by a single nucleotide change in the *qSH1* gene. As such, CRISPR/Cas9 or base editing could potentially be applied in a variety of weed species to re-create the single nucleotide polymorphism (SNP) in the homologous regulatory region of the *qSH1* gene in rice to emulate loss of shattering.

### Root Exudate Profile Modification

Natural phenomenon such as allelopathy may give insight into alternative methods for weed control. In these cases, plants are in direct chemical-mediated competition with each other, and there is a potential to exploit these natural systems to reduce weed seed banks. The alteration of the root exudate profiles in crops *via* molecular approaches to boost crop competitiveness and decrease weed fitness presents a relatively unexplored area of research for weed management. One example where this has already been achieved is in the competition of sorghum and the parasitic plant *Striga hermonthica* (Bellis et al., 2020), which is a major concern when growing this staple food throughout Africa (Ejeta and Gressel, 2007). Bellis et al. (2020) used CRISPR/Cas9 to edit a *Striga*-susceptible sorghum variety to generate a loss-of-function mutation in the *LOW GERMINATION STIMULANT 1* (*LGS1*) gene, which is believed to alter the stereochemistry of strigolactones in the root exudates, which in turn affect the fitness of the parasitic weed. Similarly, Bari et al. (2021) demonstrated CRISPR/Cas9 editing on the strigolactone biosynthetic gene, *More Axillary Growth 1* (*MAX1*), in tomato to confer resistance against root parasitic weed *Phelipanche aegyptiaca*.

Crop allelopathy and other allelopathy applications, such as straw mulching, can be effectively used to control weeds in the field (Iqbal et al., 2007; Schulz et al., 2013; Andrew et al., 2015). Identified allelochemicals include many plant secondary metabolites and plant growth regulators (Cheng and Cheng, 2015). As such, the molecular approaches discussed in this review could potentially be employed to target biosynthesis and regulatory genes of these allelochemical compounds, with the possibility to customize crop root exudate profiles that can exert negative effects on the growth of neighboring weed species. Successful implementation of this approach would be akin to the engineered crop producing its own “herbicide” to control weeds.

## Conclusion and Future Directions

Research efforts in weed science have been mainly focused on chemical weed control and herbicide resistance. Due to the lack of economic value in studying weeds (aside from studying how we can effectively kill them in farmlands) as compared to food and industrial crops, there is a general lack of weed genomic resources available, which could potentially be tapped for various purposes. The availability of weed genomic resources could aid in the further understanding of weeds’ resilience and their stress tolerance, their evolution and adaptation to various climates, and discovery of potentially untapped useful bioproducts. The advancement of biotechnological tools and their uses in weed species would directly benefit other applications. For example, we could improve our understanding of the underlying genetics of weed species and use this knowledge to boost weed growth in harsh environments for bioremediation purposes in contaminated mining sites.

Although many of the molecular approaches discussed in this review possess several technical and regulatory challenges of their own, their potential usefulness in weed management in reducing or circumventing the use of chemicals in farmlands brings many benefits. However, several roadblocks need to be addressed. Apart from the investment required for establishing transformation systems in weeds, good scientific education for the public on the use of these technologies are also required for the successful adoption of these technologies in weed management. Also, concerns regarding the use and release of gene drives into weed population, such as the unintentional transmission of genetic materials to closely related non-weed species, and the possible outcome of population collapse or extinction in weed species and the effect on the ecological scale would also need to be addressed.

## Author Contributions

AW and BC developed the concept for the review. AW and KM wrote the manuscript. YL, JH, and BC provided substantial additions and revised the manuscript. AW performed the submission of the article. All authors contributed to the article and approved the submitted version.

## Conflict of Interest

The authors declare that the research was conducted in the absence of any commercial or financial relationships that could be construed as a potential conflict of interest.

## Publisher’s Note

All claims expressed in this article are solely those of the authors and do not necessarily represent those of their affiliated organizations, or those of the publisher, the editors and the reviewers. Any product that may be evaluated in this article, or claim that may be made by its manufacturer, is not guaranteed or endorsed by the publisher.

## Footnotes

^1^ https://www.weedgenomics.org/

## References

[ref1] Abou-Khater L. Maalouf F. Jighly A. Alsamman A. M. Rubiales D. Rispail N.. (2022). Genomic regions associated with herbicide tolerance in a worldwide faba bean (*Vicia faba* L.) collection. Sci. Rep. 12:158. doi: 10.1038/s41598-021-03861-0, PMID: 34996977 PMC8741826

[ref2] Adhikari P. Goodrich E. Fernandes S. B. Lipka A. E. Tranel P. Brown P.. (2020). Genetic variation associated with PPO-inhibiting herbicide tolerance in sorghum. PLoS One 15:e0233254. doi: 10.1371/journal.pone.0233254, PMID: 33052910 PMC7556536

[ref3] Adu-Yeboah P. Malone J. M. Fleet B. Gill G. Preston C. (2020). EPSPS gene amplification confers resistance to glyphosate resistant populations of Hordeum glaucum Stued (northern barley grass) in South Australia. Pest Manag. Sci. 76, 1214–1221. doi: 10.1002/ps.5671, PMID: 31686435

[ref4] Alagbo O. Akinyemiju O. Chauhan B. (2022). Weed management in rainfed upland rice fields under varied agro-ecologies in Nigeria. Rice Sci. 29:2.

[ref5] Alphey L. S. Crisanti A. Randazzo F. Akbari O. S. (2020). Standardizing the definition of gene drive. Proc. Natl. Acad. Sci. U. S. A. 117, 30864–30867. doi: 10.1073/pnas.2020417117, PMID: 33208534 PMC7733814

[ref6] Andrew I. Storkey J. Sparkes D. (2015). A review of the potential for competitive cereal cultivars as a tool in integrated weed management. Weed Res. 55, 239–248. doi: 10.1111/wre.12137, PMID: 27478257 PMC4950144

[ref7] Araki T. Kobayashi Y. Kaya H. Iwabuchi M. (1998). The flowering-time geneFT and regulation of flowering in *Arabidopsis*. J. Plant Res. 111, 277–281. doi: 10.1007/BF02512184

[ref8] Bari V. K. Nassar J. A. Aly R. (2021). CRISPR/Cas9 mediated mutagenesis of MORE AXILLARY GROWTH 1 in tomato confers resistance to root parasitic weed Phelipanche aegyptiaca. Sci. Rep. 11:3905. doi: 10.1038/s41598-021-82897-8, PMID: 33594101 PMC7887253

[ref9] Barrett L. G. Legros M. Kumaran N. Glassop D. Raghu S. Gardiner D. M. (2019). Gene drives in plants: opportunities and challenges for weed control and engineered resilience. Proc. R. Soc. B Biol. Sci. 286:20191515. doi: 10.1098/rspb.2019.1515, PMID: 31551052 PMC6784734

[ref10] Barry G. F. Kishore G. M. Padgette S. R. Stallings W. C. (1997). Glyphosate-tolerant 5-enolpyruvylshikimate-3-phosphate synthases. US Patent. 5633435.

[ref11] Baskin C. C. Baskin J. M. (2006). The natural history of soil seed banks of arable land. Weed Sci. 54, 549–557. doi: 10.1614/WS-05-034R.1

[ref12] Basu C. Halfhill M. D. Mueller T. C. Stewart C. N. (2004). Weed genomics: new tools to understand weed biology. Trends Plant Sci. 9, 391–398. doi: 10.1016/j.tplants.2004.06.003, PMID: 15358270

[ref13] Bawa K. S. Beach J. H. (1981). Evolution of sexual systems in flowering plants. Annal. Miss. Bot. Gard. 68, 254–274. doi: 10.2307/2398798

[ref14] Bellis E. S. Kelly E. A. Lorts C. M. Gao H. Deleo V. L. Rouhan G.. (2020). Genomics of sorghum local adaptation to a parasitic plant. Proc. Natl. Acad. Sci. U. S. A. 117, 4243–4251. doi: 10.1073/pnas.1908707117, PMID: 32047036 PMC7049153

[ref15] Bernasconi G. Antonovics J. Biere A. Charlesworth D. Delph L. F. Filatov D.. (2009). Silene as a model system in ecology and evolution. Heredity 103, 5–14. doi: 10.1038/hdy.2009.34, PMID: 19367316

[ref16] Bharamappanavara M. Siddaiah A. M. Ponnuvel S. Ramappa L. Patil B. Appaiah M.. (2020). Mapping QTL hotspots associated with weed competitive traits in backcross population derived from *Oryza sativa* L. and *O. glaberrima* Steud. Sci. Rep. 10:22103. doi: 10.1038/s41598-020-78675-7, PMID: 33328509 PMC7744529

[ref17] Brunharo C. A. C. G. Hanson B. D. (2017). Vacuolar sequestration of paraquat is involved in the resistance mechanism in *Lolium perenne* L. spp. multiflorum. Front. Plant Sci. 8:1485. doi: 10.3389/fpls.2017.01485, PMID: 28890724 PMC5575147

[ref18] Burt A. (2003). Site-specific selfish genes as tools for the control and genetic engineering of natural populations. Proc. R. Soc. Lond. Ser. B Biol. Sci. 270, 921–928. doi: 10.1098/rspb.2002.2319, PMID: 12803906 PMC1691325

[ref19] Burt A. Trivers R. (2006). Genes in Conflict: The Biology of Selfish Genetic Elements. Cambridge, MA: The Belknap Press of Harvard University Press.

[ref20] Cagliari D. Dias N. P. Galdeano D. M. Dos Santos E. A. Smagghe G. Zotti M. J. (2019). Management of pest insects and plant diseases by non-transformative RNAi. Front. Plant Sci. 10:1319. doi: 10.3389/fpls.2019.01319, PMID: 31708946 PMC6823229

[ref21] Campe R. Hollenbach E. Kämmerer L. Hendriks J. Höffken H. W. Kraus H.. (2018). A new herbicidal site of action: cinmethylin binds to acyl-ACP thioesterase and inhibits plant fatty acid biosynthesis. Pestic. Biochem. Physiol. 148, 116–125. doi: 10.1016/j.pestbp.2018.04.006, PMID: 29891362

[ref22] Cardina J. Herms C. P. Doohan D. J. (2002). Crop rotation and tillage system effects on weed seedbanks. Weed Sci. 50, 448–460. doi: 10.1614/0043-1745(2002)050[0448:CRATSE]2.0.CO;2

[ref23] Carmona R. (1992). Problematic and management of weed seed banks in agricultural soils. Plant. Daninha 10, 5–16.

[ref24] Čermák T. Baltes N. J. Čegan R. Zhang Y. Voytas D. F. (2015). High-frequency, precise modification of the tomato genome. Genome Biol. 16:232. doi: 10.1186/s13059-015-0796-9, PMID: 26541286 PMC4635538

[ref25] Chan Y.-S. Huen D. S. Glauert R. Whiteway E. Russell S. (2013). Optimising homing endonuclease gene drive performance in a semi-refractory species: the *Drosophila melanogaster* experience. PLoS One 8:e54130. doi: 10.1371/journal.pone.0054130, PMID: 23349805 PMC3548849

[ref26] Charlesworth D. (2002). Plant sex determination and sex chromosomes. Heredity 88, 94–101. doi: 10.1038/sj.hdy.6800016, PMID: 11932767

[ref27] Charlesworth D. (2013). Plant sex chromosome evolution. J. Exp. Bot. 64, 405–420. doi: 10.1093/jxb/ers322, PMID: 23125359

[ref28] Chauhan B. S. (2020). Grand challenges in weed management. Front. Agron. 1:3. doi: 10.3389/fagro.2019.00003

[ref29] Chauhan B. S. Manalil S. (2022). Seedbank persistence of four summer grass weed species in the northeast cropping region of Australia. PLoS One 17:e0262288. doi: 10.1371/journal.pone.0262288, PMID: 34982794 PMC8726505

[ref30] Chen K. Wang Y. Zhang R. Zhang H. Gao C. (2019). CRISPR/Cas genome editing and precision plant breeding in agriculture. Annu. Rev. Plant Biol. 70, 667–697. doi: 10.1146/annurev-arplant-050718-100049, PMID: 30835493

[ref31] Cheng F. Cheng Z. (2015). Research progress on the use of plant allelopathy in agriculture and the physiological and ecological mechanisms of allelopathy. Front. Plant Sci. 6:1020. doi: 10.3389/fpls.2015.01020, PMID: 26635845 PMC4647110

[ref32] Chronopoulou E. Georgakis N. Nianiou-Obeidat I. Madesis P. Perperopoulou F. Pouliou F.. (2017). “Plant glutathione transferases in abiotic stress response and herbicide resistance,” in Glutathione in Plant Growth, Development, and Stress Tolerance. eds. Hossain M. A. Mostofa M. G. Diaz-Vivancos P. Burritt D. J. Fujita M. Tran L.-S. P. (Cham: Springer International Publishing).

[ref33] Coleman R. K. Gill G. S. Rebetzke G. J. (2001). Identification of quantitative trait loci for traits conferring weed competitiveness in wheat (*Triticum aestivum* L.). Aust. J. Agric. Res. 52, 1235–1246. doi: 10.1071/AR01055

[ref34] Conte S. S. Lloyd A. M. (2011). Exploring multiple drug and herbicide resistance in plants--spotlight on transporter proteins. Plant Sci. 180, 196–203. doi: 10.1016/j.plantsci.2010.10.015, PMID: 21421361

[ref35] Croser J. Mao D. Dron N. Michelmore S. Mcmurray L. Preston C.. (2021). Evidence for the application of emerging technologies to accelerate crop improvement—a collaborative pipeline to introgress herbicide tolerance into chickpea. Front. Plant Sci. 12:779122. doi: 10.3389/fpls.2021.779122, PMID: 34925421 PMC8678039

[ref36] Cummins I. Wortley D. J. Sabbadin F. He Z. Coxon C. R. Straker H. E.. (2013). Key role for a glutathione transferase in multiple-herbicide resistance in grass weeds. Proc. Natl. Acad. Sci. U. S. A. 110, 5812–5817. doi: 10.1073/pnas.1221179110, PMID: 23530204 PMC3625300

[ref37] Da Costa T. P. S. Hall C. J. Panjikar S. Wyllie J. A. Christoff R. M. Bayat S.. (2021). Towards novel herbicide modes of action by inhibiting lysine biosynthesis in plants. Elife 10:e69444. doi: 10.7554/eLife.69444, PMID: 34313586 PMC8341977

[ref38] Dayan F. E. (2019). Current status and future prospects in herbicide discovery. Plants 8:341. doi: 10.3390/plants8090341, PMID: 31514265 PMC6783942

[ref39] Dayan F. E. Haesaert G. Van Leeuwen T. Holden-Dye L. Crossthwaite A. Nauen R. (2019). Pesticides modes of action and resistance: a perspective from the 2019 IUPAC congress. Outlooks Pest Manag. 30, 157–163. doi: 10.1564/v30_aug_04

[ref40] De Vida F. B. P. Laca E. A. Mackill D. J. Fernández G. M. Fischer A. J. (2006). Relating rice traits to weed competitiveness and yield: a path analysis. Weed Sci. 54, 1122–1131. doi: 10.1614/WS-06-042R.1

[ref41] Debeaujon I. Lepiniec L. Pourcel L. Routaboul J.-M. (2007). “Seed coat development and dormancy,” in Annual Plant Reviews, Seed Development, Dormancy and Germination. eds. Bradford K. Nonogaki H. (UK: Blackwell Publishing Ltd).

[ref42] Délye C. (2013). Unravelling the genetic bases of non-target-site-based resistance (NTSR) to herbicides: a major challenge for weed science in the forthcoming decade. Pest Manag. Sci. 69, 176–187. doi: 10.1002/ps.3318, PMID: 22614948

[ref43] Dicarlo J. E. Chavez A. Dietz S. L. Esvelt K. M. Church G. M. (2015). Safeguarding CRISPR-Cas9 gene drives in yeast. Nat. Biotechnol. 33, 1250–1255. doi: 10.1038/nbt.3412, PMID: 26571100 PMC4675690

[ref44] Dimaano N. G. B. Ali J. Mahender A. Sta Cruz P. C. Baltazar A. M. Diaz M. G. Q.. (2020). Identification of quantitative trait loci governing early germination and seedling vigor traits related to weed competitive ability in rice. Euphytica 216:159. doi: 10.1007/s10681-020-02694-8, PMID: 33029032 PMC7510932

[ref45] Dixon R. A. Paiva N. L. (1995). Stress-induced phenylpropanoid metabolism. Plant Cell 7, 1085–1097. doi: 10.1105/tpc.7.7.1085, PMID: 12242399 PMC160915

[ref46] Domagalska M. A. Leyser O. (2011). Signal integration in the control of shoot branching. Nat. Rev. Mol. Cell Biol. 12, 211–221. doi: 10.1038/nrm3088, PMID: 21427763

[ref47] Dong Y. Wang Y.-Z. (2015). Seed shattering: from models to crops. Front. Plant Sci. 6:476. doi: 10.3389/fpls.2015.00476, PMID: 26157453 PMC4478375

[ref48] Duke S. O. (2012). Why have no new herbicide modes of action appeared in recent years? Pest Manag. Sci. 68, 505–512. doi: 10.1002/ps.2333, PMID: 22190296

[ref49] Edwards K. Cramer C. L. Bolwell G. P. Dixon R. A. Schuch W. Lamb C. J. (1985). Rapid transient induction of phenylalanine ammonia-lyase mRNA in elicitor-treated bean cells. Proc. Natl. Acad. Sci. U. S. A. 82, 6731–6735. doi: 10.1073/pnas.82.20.6731, PMID: 16593613 PMC390760

[ref50] Ejeta G. Gressel J. (2007). Integrating New Technologies for Striga Control: Towards Ending the Witch-Hunt. Singapore: World Scientific Publishing Co Pte Ltd.

[ref51] Elgersma A. Leeuwangh J. Wilms H. J. (1988). Abscission and seed shattering in perennial ryegrass (*Lolium perenne* L.). Euphytica 39, 51–57. doi: 10.1007/BF00043367

[ref52] Faure S. Higgins J. Turner A. Laurie D. A. (2007). The FLOWERING LOCUS T-like gene family in barley (Hordeum vulgare). Genetics 176, 599–609. doi: 10.1534/genetics.106.069500, PMID: 17339225 PMC1893030

[ref53] Feng Y. Huang Q. Zhang R. Li J. Luo K. Chen Y. (2022). Molecular characterisation of PAL gene family reveals their role in abiotic stress response in lucerne (*Medicago sativa*). Crop Past. Sci. 73:1558. doi: 10.1071/CP21558

[ref54] Finch-Savage W. E. Leubner-Metzger G. (2006). Seed dormancy and the control of germination. New Phytol. 171, 501–523. doi: 10.1111/j.1469-8137.2006.01787.x, PMID: 16866955

[ref55] Foley M. E. Fennimore S. A. (1998). Genetic basis for seed dormancy. Seed Sci. Res. 8, 173–182.

[ref56] Fuller D. Q. Allaby R. (2009). “Seed dispersal and crop domestication: shattering, germination and seasonality in evolution under cultivation,” in Annual Plant Reviews Volume 38: Fruit Development and Seed Dispersal. (UK: Blackwell Publishing Ltd).

[ref57] Gaines T. A. Duke S. O. Morran S. Rigon C. A. G. Tranel P. J. Küpper A.. (2020). Mechanisms of evolved herbicide resistance. J. Biol. Chem. 295, 10307–10330. doi: 10.1074/jbc.REV120.013572, PMID: 32430396 PMC7383398

[ref58] Gaines T. A. Zhang W. Wang D. Bukun B. Chisholm S. T. Shaner D. L.. (2010). Gene amplification confers glyphosate resistance in *Amaranthus palmeri*. Proc. Natl. Acad. Sci. U. S. A. 107, 1029–1034. doi: 10.1073/pnas.0906649107, PMID: 20018685 PMC2824275

[ref59] Gallavotti A. (2013). The role of auxin in shaping shoot architecture. J. Exp. Bot. 64, 2593–2608. doi: 10.1093/jxb/ert141, PMID: 23709672

[ref60] Gantz V. M. Bier E. (2015). Genome editing. The mutagenic chain reaction: a method for converting heterozygous to homozygous mutations. Science 348, 442–444. doi: 10.1126/science.aaa5945, PMID: 25908821 PMC4687737

[ref61] Gantz V. M. Jasinskiene N. Tatarenkova O. Fazekas A. Macias V. M. Bier E.. (2015). Highly efficient Cas9-mediated gene drive for population modification of the malaria vector mosquito *Anopheles stephensi*. Proc. Natl. Acad. Sci. U. S. A. 112, E6736–E6743. doi: 10.1073/pnas.1521077112, PMID: 26598698 PMC4679060

[ref62] Ge X. D’avignon D. A. Ackerman J. J. H. Collavo A. Sattin M. Ostrander E. L.. (2012). Vacuolar glyphosate-sequestration correlates with glyphosate resistance in ryegrass (Lolium spp.) from Australia, South America, and Europe: a 31P NMR investigation. J. Agric. Food Chem. 60, 1243–1250. doi: 10.1021/jf203472s, PMID: 22224711

[ref63] Ge X. D’avignon D. A. Ackerman J. J. Duncan B. Spaur M. B. Sammons R. D. (2011). Glyphosate-resistant horseweed made sensitive to glyphosate: low-temperature suppression of glyphosate vacuolar sequestration revealed by 31P NMR. Pest Manag. Sci. 67, 1215–1221. doi: 10.1002/ps.2169, PMID: 21495156

[ref64] Gil-Humanes J. Wang Y. Liang Z. Shan Q. Ozuna C. V. Sánchez-León S.. (2017). High-efficiency gene targeting in hexaploid wheat using DNA replicons and CRISPR/Cas9. Plant J. 89, 1251–1262. doi: 10.1111/tpj.13446, PMID: 27943461 PMC8439346

[ref65] Goggin D. E. Cawthray G. R. Powles S. B. (2016). 2,4-D resistance in wild radish: reduced herbicide translocation via inhibition of cellular transport. J. Exp. Bot. 67, 3223–3235. doi: 10.1093/jxb/erw120, PMID: 26994475 PMC4892717

[ref66] Graeber K. Nakabayashi K. Miatton E. Leubner-Metzger G. Soppe W. J. (2012). Molecular mechanisms of seed dormancy. Plant Cell Environ. 35, 1769–1786. doi: 10.1111/j.1365-3040.2012.02542.x, PMID: 22620982

[ref67] Grunwald H. A. Gantz V. M. Poplawski G. Xu X. S. Bier E. Cooper K. L. (2019). Super-Mendelian inheritance mediated by CRISPR-Cas9 in the female mouse germline. Nature 566, 105–109. doi: 10.1038/s41586-019-0875-2, PMID: 30675057 PMC6367021

[ref68] Gu X.-Y. Pipatpongpinyo W. Zhang L. Zhou Y. Ye H. Feng J. (2018). Two contrasting patterns and underlying genes for coadaptation of seed dormancy and flowering time in rice. Sci. Rep. 8:16813. doi: 10.1038/s41598-018-34850-5, PMID: 30429528 PMC6235893

[ref69] Hammond A. Galizi R. Kyrou K. Simoni A. Siniscalchi C. Katsanos D.. (2016). A CRISPR-Cas9 gene drive system targeting female reproduction in the malaria mosquito vector Anopheles gambiae. Nat. Biotechnol. 34, 78–83. doi: 10.1038/nbt.3439, PMID: 26641531 PMC4913862

[ref70] Hanzawa Y. Money T. Bradley D. (2005). A single amino acid converts a repressor to an activator of flowering. Proc. Natl. Acad. Sci. U. S. A. 102, 7748–7753. doi: 10.1073/pnas.0500932102, PMID: 15894619 PMC1140427

[ref71] Harker K. N. O’donovan J. T. (2013). Recent weed control, weed management, and integrated weed management. Weed Technol. 27, 1–11. doi: 10.1614/WT-D-12-00109.1, PMID: 33788411

[ref72] Harlan J. R. Dewet J. M. (1965). Some thoughts about weeds. Econ. Bot. 19, 16–24. doi: 10.1007/BF02971181, PMID: 35069781

[ref73] Hayama R. Coupland G. (2004). The molecular basis of diversity in the photoperiodic flowering responses of *Arabidopsis* and rice. Plant Physiol. 135, 677–684. doi: 10.1104/pp.104.042614, PMID: 15208414 PMC514104

[ref74] Heap I. (2022). The International Herbicide-Resistant Weed Database [Online]. Available at: https://www.weedscience.org/Pages/ChronologicalIncrease.aspx (Accessed February 3, 2022).

[ref75] Hirose S. Kawahigashi H. Tagiri A. Imaishi H. Ohkawa H. Ohkawa Y. (2007). Tissue-specific expression of rice CYP72A21 induced by auxins and herbicides. Plant Biotechnol. Rep. 1, 27–36. doi: 10.1007/s11816-006-0003-2

[ref76] Huang J. Gu M. Lai Z. Fan B. Shi K. Zhou Y.-H.. (2010). Functional analysis of the *Arabidopsis* PAL gene family in plant growth, development, and response to environmental stress. Plant Physiol. 153, 1526–1538. doi: 10.1104/pp.110.157370, PMID: 20566705 PMC2923909

[ref77] Huang T.-K. Puchta H. (2019). CRISPR/Cas-mediated gene targeting in plants: finally a turn for the better for homologous recombination. Plant Cell Rep. 38, 443–453. doi: 10.1007/s00299-019-02379-0, PMID: 30673818

[ref78] Iqbal J. Cheema Z. An M. (2007). Intercropping of field crops in cotton for the management of purple nutsedge (*Cyperus rotundus* L.). Plant Soil 300, 163–171. doi: 10.1007/s11104-007-9400-8

[ref79] Izawa T. Oikawa T. Sugiyama N. Tanisaka T. Yano M. Shimamoto K. (2002). Phytochrome mediates the external light signal to repress FT orthologs in photoperiodic flowering of rice. Genes Dev. 16, 2006–2020. doi: 10.1101/gad.999202, PMID: 12154129 PMC186415

[ref80] Jabran K. Chauhan B. S. (eds.) (2018). “Overview and significance of non-chemical weed control,” in Non-Chemical Weed Control (London, United Kingdom: Academic Press).

[ref81] Jóri B. Soós V. Szegő D. Páldi E. Szigeti Z. Rácz I.. (2007). Role of transporters in paraquat resistance of horseweed *Conyza canadensis* (L.) Cronq. Pestic. Biochem. Physiol. 88, 57–65. doi: 10.1016/j.pestbp.2006.08.013

[ref82] Jugulam M. Shyam C. (2019). Non-target-site resistance to herbicides: recent developments. Plants 8:417. doi: 10.3390/plants8100417, PMID: 31618956 PMC6843234

[ref83] Kim D. S. Hwang B. K. (2014). An important role of the pepper phenylalanine ammonia-lyase gene (PAL1) in salicylic acid-dependent signalling of the defence response to microbial pathogens. J. Exp. Bot. 65, 2295–2306. doi: 10.1093/jxb/eru109, PMID: 24642849 PMC4036500

[ref84] Kim W. Park T. I. Yoo S. J. Jun A. R. Ahn J. H. (2013). Generation and analysis of a complete mutant set for the *Arabidopsis* FT/TFL1 family shows specific effects on thermo-sensitive flowering regulation. J. Exp. Bot. 64, 1715–1729. doi: 10.1093/jxb/ert036, PMID: 23404901 PMC3617836

[ref85] Klupczyńska E. A. Pawłowski T. A. (2021). Regulation of seed dormancy and germination mechanisms in a changing environment. Int. J. Mol. Sci. 22:1357. doi: 10.3390/ijms22031357, PMID: 33572974 PMC7866424

[ref86] Knezevic S. Z. Jhala A. Datta A. (2017). “Integrated weed management,” in Encyclopedia of Applied Plant Sciences. eds. Thomas B. Murray B. G. Murphy D. J. (Oxford: Academic Press).

[ref87] Kobayashi Y. Kaya H. Goto K. Iwabuchi M. Araki T. (1999). A pair of related genes with antagonistic roles in mediating flowering signals. Science 286, 1960–1962. doi: 10.1126/science.286.5446.1960, PMID: 10583960

[ref88] Konishi S. Izawa T. Lin S. Y. Ebana K. Fukuta Y. Sasaki T.. (2006). An SNP caused loss of seed shattering during rice domestication. Science 312, 1392–1396. doi: 10.1126/science.1126410, PMID: 16614172

[ref89] Koornneef M. Hanhart C. J. Van Der Veen J. H. (1991). A genetic and physiological analysis of late flowering mutants in *Arabidopsis thaliana*. Mol. Gen. Genet. 229, 57–66. doi: 10.1007/BF00264213, PMID: 1896021

[ref90] Kyrou K. Hammond A. M. Galizi R. Kranjc N. Burt A. Beaghton A. K.. (2018). A CRISPR-Cas9 gene drive targeting doublesex causes complete population suppression in caged Anopheles gambiae mosquitoes. Nat. Biotechnol. 36, 1062–1066. doi: 10.1038/nbt.4245, PMID: 30247490 PMC6871539

[ref91] Laforest M. Soufiane B. Simard M.-J. Obeid K. Page E. Nurse R. E. (2017). Acetyl-CoA carboxylase overexpression in herbicide-resistant large crabgrass (*Digitaria sanguinalis*). Pest Manag. Sci. 73, 2227–2235. doi: 10.1002/ps.4675, PMID: 28755464

[ref92] Lewellyn R. S. Ronning D. Ouzman J. Walker S. Mayfield A. Clarke M. (2016). Impact of weeds on Australian grain production: the cost of weeds to Australian grain growers and the adoption of weed management and tillage practices. Report for GRDC. CSIRO, Australia.

[ref93] Li B. Foley M. E. (1997). Genetic and molecular control of seed dormancy. Trends Plant Sci. 2, 384–389. doi: 10.1016/S1360-1385(97)90053-4

[ref94] Li F. Numa H. Hara N. Sentoku N. Ishii T. Fukuta Y.. (2019). Identification of a locus for seed shattering in rice (*Oryza sativa* L.) by combining bulked segregant analysis with whole-genome sequencing. Mol. Breed. 39, 1–14. doi: 10.1007/s11032-019-0941-3

[ref95] Lin Z. Li X. Shannon L. M. Yeh C.-T. Wang M. L. Bai G.. (2012). Parallel domestication of the Shattering1 genes in cereals. Nat. Genet. 44, 720–724. doi: 10.1038/ng.2281, PMID: 22581231 PMC3532051

[ref96] Lindholm A. K. Dyer K. A. Firman R. C. Fishman L. Forstmeier W. Holman L.. (2016). The ecology and evolutionary dynamics of meiotic drive. Trends Ecol. Evol. 31, 315–326. doi: 10.1016/j.tree.2016.02.001, PMID: 26920473

[ref97] Lombardo L. (2014). Genetic use restriction technologies: a review. Plant Biotechnol. J. 12, 995–1005. doi: 10.1111/pbi.12242, PMID: 25185773

[ref98] Lu H.-P. Edwards M. Wang Q.-Z. Zhao H.-J. Fu H.-W. Huang J.-Z.. (2015). Expression of cytochrome P450 CYP81A6 in rice: tissue specificity, protein subcellular localization, and response to herbicide application. J. Zhejiang Univ. Sci. B 16, 113–122. doi: 10.1631/jzus.B1400168, PMID: 25644466 PMC4322422

[ref99] Lv S. Wu W. Wang M. Meyer R. S. Ndjiondjop M.-N. Tan L.. (2018). Genetic control of seed shattering during African rice domestication. Nat. Plants 4, 331–337. doi: 10.1038/s41477-018-0164-3, PMID: 29872176

[ref100] Manalil S. Chauhan B. S. (2021). Seedbank persistence and emergence pattern of Argemone mexicana, Rapistrum rugosum and Sonchus oleraceus in the eastern grain region of Australia. Sci. Rep. 11:18095. doi: 10.1038/s41598-021-97614-8, PMID: 34508123 PMC8433185

[ref101] Manning A. (2021). International Weed Genomics Consortium—Collaborative Effort to Combat Crop-Threatening Weeds Headed by CSU Scientists. Available at: https://agsci.source.colostate.edu/international-effort-to-combat-crop-threatening-weeds-headed-by-csu-scientists/ (Accessed October 22, 2021).

[ref102] Martin S. L. Parent J.-S. Laforest M. Page E. Kreiner J. M. James T. (2019). Population genomic approaches for weed science. Plants 8:354. doi: 10.3390/plants8090354, PMID: 31546893 PMC6783936

[ref103] Martinoia E. Grill E. Tommasini R. Kreuz K. Amrhein N. (1993). ATP-dependent glutathione S-conjugate ‘export’ pump in the vacuolar membrane of plants. Nature 364, 247–249. doi: 10.1038/364247a0

[ref104] Massel K. Lam Y. Wong A. C. S. Hickey L. T. Borrell A. K. Godwin I. D. (2021). Hotter, drier, CRISPR: the latest edit on climate change. Theor. Appl. Genet. 134, 1691–1709. doi: 10.1007/s00122-020-03764-0, PMID: 33420514

[ref105] Mcmillan H. E. Liu G. Shelton H. M. Dalzell S. A. Godwin I. D. Gamage H.. (2019). Sterile leucaena becomes a reality? Trop. Grasslands-Forrajes Trop. 7, 74–79. doi: 10.17138/tgft(7)74-79

[ref106] Miki D. Zhang W. Zeng W. Feng Z. Zhu J.-K. (2018). CRISPR/Cas9-mediated gene targeting in *Arabidopsis* using sequential transformation. Nat. Commun. 9:1967. doi: 10.1038/s41467-018-04416-0, PMID: 29773790 PMC5958078

[ref107] Mitter N. Worrall E. A. Robinson K. E. Li P. Jain R. G. Taochy C.. (2017). Clay nanosheets for topical delivery of RNAi for sustained protection against plant viruses. Nat. Plants 3:16207. doi: 10.1038/nplants.2016.207 28067898

[ref108] Mizutani M. (2012). Impacts of diversification of cytochrome P450 on plant metabolism. Biol. Pharm. Bull. 35, 824–832. doi: 10.1248/bpb.35.824, PMID: 22687470

[ref109] Molla K. A. Sretenovic S. Bansal K. C. Qi Y. (2021). Precise plant genome editing using base editors and prime editors. Nat. Plants 7, 1166–1187. doi: 10.1038/s41477-021-00991-1, PMID: 34518669

[ref110] Monéger F. (2007). Sex determination in plants. Plant Signal. Behav. 2, 178–179. doi: 10.4161/psb.2.3.3728, PMID: 19704689 PMC2634050

[ref111] Montgomery J. S. Giacomini D. A. Weigel D. Tranel P. J. (2021). Male-specific Y-chromosomal regions in waterhemp (*Amaranthus tuberculatus*) and Palmer amaranth (*Amaranthus palmeri*). New Phytol. 229, 3522–3533. doi: 10.1111/nph.17108, PMID: 33301599

[ref112] Montgomery J. S. Sadeque A. Giacomini D. A. Brown P. J. Tranel P. J. (2019). Sex-specific markers for waterhemp (*Amaranthus tuberculatus*) and Palmer amaranth (*Amaranthus palmeri*). Weed Sci. 67, 412–418. doi: 10.1017/wsc.2019.27

[ref113] Moretti M. L. Hanson B. D. (2017). Reduced translocation is involved in resistance to glyphosate and paraquat in Conyza bonariensis and Conyza canadensis from California. Weed Res. 57, 25–34. doi: 10.1111/wre.12230

[ref114] National Academies of Sciences, Engineering, and Medicine (2016). Gene Drives on the Horizon: Advancing Science, Navigating Uncertainty, and Aligning Research With Public Values, Washington, DC: The National Academies Press. 27536751

[ref115] Neve P. (2018). Gene drive systems: do they have a place in agricultural weed management? Pest Manag. Sci. 74, 2671–2679. doi: 10.1002/ps.5137, PMID: 29999229 PMC6282749

[ref116] Newhouse K. E. Smith W. A. Starrett M. A. Schaefer T. J. Singh B. K. (1992). Tolerance to imidazolinone herbicides in wheat. Plant Physiol. 100, 882–886. doi: 10.1104/pp.100.2.882, PMID: 16653071 PMC1075639

[ref117] Ngo T. D. Malone J. M. Boutsalis P. Gill G. Preston C. (2018). EPSPS gene amplification conferring resistance to glyphosate in windmill grass (*Chloris truncata*) in Australia. Pest Manag. Sci. 74, 1101–1108. doi: 10.1002/ps.4573, PMID: 28317250

[ref118] Nonogaki H. (2014). Seed dormancy and germination—emerging mechanisms and new hypotheses. Front. Plant Sci. 5:223. doi: 10.3389/fpls.2014.00233, PMID: 24904627 PMC4036127

[ref119] OECD/FAO (2021). Agricultural and Food Markets: Trends and Prospects. OECD-FAO Agricultural Outlook 2021–2030. Paris: OECD Publishing.

[ref120] Oerke E. C. (2006). Crop losses to pests. J. Agric. Sci. 144, 31–43. doi: 10.1017/S0021859605005708, PMID: 35366099

[ref121] Padgette S. R. Kolacz K. H. Delannay X. Re D. B. Lavallee B. J. Tinius C. N.. (1995). Development, identification, and characterization of a glyphosate-tolerant soybean line. Crop Sci. 35, 1451–1461. doi: 10.2135/cropsci1995.0011183X003500050032x

[ref122] Padgette S. Re D. Barry G. Eichholtz D. Delannay X. Fuchs R.. (1996). “New weed control opportunities: development of soybeans with a roundup ready gene,” in Herbicide Resistant Crops: Agricultural, Economic, Environmental, Regulatory, and Technological Aspects. ed. Stephen D. (Boca Raton, FL: CRC Press).

[ref123] Pasquer F. Ochsner U. Zarn J. Keller B. (2006). Common and distinct gene expression patterns induced by the herbicides 2,4-dichlorophenoxyacetic acid, cinidon-ethyl and tribenuron-methyl in wheat. Pest Manag. Sci. 62, 1155–1167. doi: 10.1002/ps.1291, PMID: 17054088

[ref124] Patterson E. L. Saski C. Küpper A. Beffa R. Gaines T. A. (2019). Omics potential in herbicide-resistant weed management. Plants 8:607. doi: 10.3390/plants8120607, PMID: 31847327 PMC6963460

[ref125] Peng F. Zhang W. Zeng W. Zhu J.-K. Miki D. (2020). Gene targeting in *Arabidopsis* via an all-in-one strategy that uses a translational enhancer to aid Cas9 expression. Plant Biotechnol. J. 18, 892–894. doi: 10.1111/pbi.13265, PMID: 31553828 PMC7061861

[ref126] Perotti V. E. Larran A. S. Palmieri V. E. Martinatto A. K. Permingeat H. R. (2020). Herbicide resistant weeds: a call to integrate conventional agricultural practices, molecular biology knowledge and new technologies. Plant Sci. 290:110255. doi: 10.1016/j.plantsci.2019.110255, PMID: 31779903

[ref127] Peters K. J. Gerowitt B. (2014). Important maize weeds profit in growth and reproduction from climate change conditions represented by higher temperatures and reduced humidity. J. Appl. Bot. Food Qual. 87, 234–242. doi: 10.5073/JABFQ.2014.087.033

[ref128] Peterson M. A. Mcmaster S. A. Riechers D. E. Skelton J. Stahlman P. W. (2016). 2,4-D past, present, and future: a review. Weed Technol. 30, 303–345. doi: 10.1614/WT-D-15-00131.1

[ref129] Pimentel D. Zuniga R. Morrison D. (2005). Update on the environmental and economic costs associated with alien-invasive species in the United States. Ecol. Econ. 52, 273–288. doi: 10.1016/j.ecolecon.2004.10.002

[ref130] Pipatpongpinyo W. Korkmaz U. Wu H. Kena A. Ye H. Feng J.. (2020). Assembling seed dormancy genes into a system identified their effects on seedbank longevity in weedy rice. Heredity 124, 135–145. doi: 10.1038/s41437-019-0253-8, PMID: 31391557 PMC6906365

[ref131] Puchta H. (2004). The repair of double-strand breaks in plants: mechanisms and consequences for genome evolution. J. Exp. Bot. 56, 1–14. doi: 10.1093/jxb/eri025, PMID: 15557293

[ref132] Putterill J. Robson F. Lee K. Simon R. Coupland G. (1995). The CONSTANS gene of *Arabidopsis* promotes flowering and encodes a protein showing similarities to zinc finger transcription factors. Cell 80, 847–857. doi: 10.1016/0092-8674(95)90288-0, PMID: 7697715

[ref133] Ramesh K. Matloob A. Aslam F. Florentine S. K. Chauhan B. S. (2017). Weeds in a changing climate: vulnerabilities, consequences, and implications for future weed management. Front. Plant Sci. 8:95. doi: 10.3389/fpls.2017.00095, PMID: 28243245 PMC5303747

[ref134] Ravet K. Patterson E. L. Krähmer H. Hamouzová K. Fan L. Jasieniuk M.. (2018). The power and potential of genomics in weed biology and management. Pest Manag. Sci. 74, 2216–2225. doi: 10.1002/ps.5048, PMID: 29687580

[ref135] Riar D. S. Burke I. C. Yenish J. P. Bell J. Gill K. (2011). Inheritance and physiological basis for 2,4-D resistance in prickly lettuce (*Lactuca serriola* L.). J. Agric. Food Chem. 59, 9417–9423. doi: 10.1021/jf2019616, PMID: 21790161

[ref136] Rong Y. S. Golic K. G. (2003). The homologous chromosome is an effective template for the repair of mitotic DNA double-strand breaks in *Drosophila*. Genetics 165, 1831–1842. doi: 10.1093/genetics/165.4.1831, PMID: 14704169 PMC1462885

[ref137] Roux F. Reboud X. (2007). Herbicide resistance dynamics in a spatially heterogeneous environment. Crop Prot. 26, 335–341. doi: 10.1016/j.cropro.2005.08.020

[ref138] Schaller G. E. Bishopp A. Kieber J. J. (2015). The Yin-Yang of hormones cytokinin and auxin interactions in plant development. Plant Cell 27, 44–63. doi: 10.1105/tpc.114.133595, PMID: 25604447 PMC4330578

[ref139] Schulz M. Marocco A. Tabaglio V. Macias F. A. Molinillo J. M. (2013). Benzoxazinoids in rye allelopathy-from discovery to application in sustainable weed control and organic farming. J. Chem. Ecol. 39, 154–174. doi: 10.1007/s10886-013-0235-x, PMID: 23385365

[ref140] Schwartz L. M. Gibson D. J. Gage K. L. Matthews J. L. Jordan D. L. Owen M. D.. (2015). Seedbank and field emergence of weeds in glyphosate-resistant cropping systems in the United States. Weed Sci. 63, 425–439. doi: 10.1614/WS-D-14-00089.1

[ref141] Schwartz-Lazaro L. M. Copes J. T. (2019). A review of the soil seedbank from a weed scientists perspective. Agronomy 9:369. doi: 10.3390/agronomy9070369

[ref142] Seymour G. B. Østergaard L. Chapman N. H. Knapp S. Martin C. (2013). Fruit development and ripening. Annu. Rev. Plant Biol. 64, 219–241. doi: 10.1146/annurev-arplant-050312-120057, PMID: 23394500

[ref143] Shi C. Zheng Y. Geng J. Liu C. Pei H. Ren Y.. (2020). Identification of herbicide resistance loci using a genome-wide association study and linkage mapping in Chinese common wheat. Crop J. 8, 666–675. doi: 10.1016/j.cj.2020.02.004

[ref144] Shino M. Hamada T. Shigematsu Y. Hirase K. Banba S. Tsukamoto Y.. (2021). “Chapter 30—discovery and mode of action of cyclopyrimorate: a new paddy rice herbicide,” in Recent Highlights in the Discovery and Optimization of Crop Protection Products. eds. Maienfisch P. Mangelinckx S. (London, United Kingdom: Academic Press).

[ref145] Sosnoskie L. M. Herms C. P. Cardina J. Webster T. M. (2009). Seedbank and emerged weed communities following adoption of glyphosate-resistant crops in a long-term tillage and rotation study. Weed Sci. 57, 261–270. doi: 10.1614/WS-08-147.1

[ref146] Stewart C. N. Tranel P. J. Horvath D. P. Anderson J. V. Rieseberg L. H. Westwood J. H.. (2009). Evolution of weediness and invasiveness: charting the course for weed genomics. Weed Sci. 57, 451–462. doi: 10.1614/WS-09-011.1

[ref147] Su Y. H. Frommer W. B. Ludewig U. (2004). Molecular and functional characterization of a family of amino acid transporters from *Arabidopsis*. Plant Physiol. 136, 3104–3113. doi: 10.1104/pp.104.045278, PMID: 15377779 PMC523371

[ref148] Székács A. (2021). “3—Herbicide mode of action,” in Herbicides. eds. Mesnage R. Zaller J. G. (Amsterdam, Netherlands: Elsevier). doi: 10.1016/B978-0-12-823674-1.00008-0

[ref149] Theodoulou F. L. (2000). Plant ABC transporters. Biochim. Biophys. Acta 1465, 79–103. doi: 10.1016/s0005-2736(00)00132-2, PMID: 10748248

[ref150] Thyssen G. Mccarty J. C. Li P. Jenkins J. N. Fang D. D. (2014). Genetic mapping of non-target-site resistance to a sulfonylurea herbicide (Envoke®) in upland cotton (*Gossypium hirsutum* L.). Mol. Breed. 33, 341–348. doi: 10.1007/s11032-013-9953-6

[ref151] Thyssen G. N. Naoumkina M. Mccarty J. C. Jenkins J. N. Florane C. Li P.. (2018). The P450 gene CYP749A16 is required for tolerance to the sulfonylurea herbicide trifloxysulfuron sodium in cotton (*Gossypium hirsutum* L.). BMC Plant Biol. 18:186. doi: 10.1186/s12870-018-1414-2, PMID: 30200872 PMC6131939

[ref152] Torti S. Schlesier R. Thümmler A. Bartels D. Römer P. Koch B.. (2021). Transient reprogramming of crop plants for agronomic performance. Nat. Plants 7, 159–171. doi: 10.1038/s41477-021-00851-y, PMID: 33594264

[ref153] Tyagi S. Kesiraju K. Saakre M. Rathinam M. Raman V. Pattanayak D.. (2020). Genome editing for resistance to insect pests: an emerging tool for crop improvement. ACS Omega 5, 20674–20683. doi: 10.1021/acsomega.0c01435, PMID: 32875201 PMC7450494

[ref154] Valderrama J. A. Kulkarni S. S. Nizet V. Bier E. (2019). A bacterial gene-drive system efficiently edits and inactivates a high copy number antibiotic resistance locus. Nat. Commun. 10:5726. doi: 10.1038/s41467-019-13649-6, PMID: 31844051 PMC6915771

[ref155] Van Etten M. Lee K. M. Chang S. M. Baucom R. S. (2020). Parallel and nonparallel genomic responses contribute to herbicide resistance in Ipomoea purpurea, a common agricultural weed. PLoS Genet. 16:e1008593. doi: 10.1371/journal.pgen.1008593, PMID: 32012153 PMC7018220

[ref156] Vanstraelen M. Benková E. (2012). Hormonal interactions in the regulation of plant development. Annu. Rev. Cell Dev. Biol. 28, 463–487. doi: 10.1146/annurev-cellbio-101011-155741, PMID: 22856461

[ref157] Vigueira C. C. Olsen K. M. Caicedo A. L. (2013). The red queen in the corn: agricultural weeds as models of rapid adaptive evolution. Heredity 110, 303–311. doi: 10.1038/hdy.2012.104, PMID: 23188175 PMC3607111

[ref158] Visser B. Van Der Meer I. Louwaars N. Beekwilder J. Eaton D. (2001). The impact of ‘terminator’ technology. Biotechnol. Dev. Monit. 48, 9–12.

[ref159] Vivian R. Silva A. A. Gimenes M. Fagan E. B. Ruiz S. T. Labonia Y. (2008). Weed seed dormancy as a survival mechanism—brief review. Planta Daninha 26, 695–706. doi: 10.1590/S0100-83582008000300026

[ref160] Vivian-Smith A. Koltunow A. M. (1999). Genetic analysis of growth-regulator-induced parthenocarpy in *Arabidopsis*. Plant Physiol. 121, 437–452. doi: 10.1104/pp.121.2.437, PMID: 10517835 PMC59406

[ref161] Vogt T. (2010). Phenylpropanoid biosynthesis. Mol. Plant 3, 2–20. doi: 10.1093/mp/ssp106, PMID: 20035037

[ref162] Waldie T. Leyser O. (2018). Cytokinin targets auxin transport to promote shoot branching. Plant Physiol. 177, 803–818. doi: 10.1104/pp.17.01691, PMID: 29717021 PMC6001322

[ref163] Wang Y. Cheng X. Shan Q. Zhang Y. Liu J. Gao C.. (2014). Simultaneous editing of three homoeoalleles in hexaploid bread wheat confers heritable resistance to powdery mildew. Nat. Biotechnol. 32, 947–951. doi: 10.1038/nbt.2969, PMID: 25038773

[ref164] Westwood J. H. Charudattan R. Duke S. O. Fennimore S. A. Marrone P. Slaughter D. C.. (2018). Weed management in 2050: perspectives on the future of weed science. Weed Sci. 66, 275–285. doi: 10.1017/wsc.2017.78

[ref165] Windbichler N. Menichelli M. Papathanos P. A. Thyme S. B. Li H. Ulge U. Y.. (2011). A synthetic homing endonuclease-based gene drive system in the human malaria mosquito. Nature 473, 212–215. doi: 10.1038/nature09937, PMID: 21508956 PMC3093433

[ref166] Wolabu T. W. Zhang F. Niu L. Kalve S. Bhatnagar-Mathur P. Muszynski M. G.. (2016). Three FLOWERING LOCUS T-like genes function as potential florigens and mediate photoperiod response in sorghum. New Phytol. 210, 946–959. doi: 10.1111/nph.13834, PMID: 26765652

[ref167] Wong A. C. Hecht V. F. Picard K. Diwadkar P. Laurie R. E. Wen J.. (2014). Isolation and functional analysis of CONSTANS-LIKE genes suggests that a central role for CONSTANS in flowering time control is not evolutionarily conserved in *Medicago truncatula*. Front. Plant Sci. 5:486. doi: 10.3389/fpls.2014.00486, PMID: 25278955 PMC4166892

[ref168] Xu L. Liu H. Kilian A. Bhoite R. Liu G. Si P.. (2020). QTL mapping using a high-density genetic map to identify candidate genes associated with metribuzin tolerance in hexaploid wheat (*Triticum aestivum* L.). Front. Plant Sci. 11:573439. doi: 10.3389/fpls.2020.573439, PMID: 33042190 PMC7527527

[ref169] Xu J. Wang X.-Y. Guo W.-Z. (2015). The cytochrome P450 superfamily: key players in plant development and defense. J. Integr. Agric. 14, 1673–1686. doi: 10.1016/S2095-3119(14)60980-1

[ref170] Yano M. Katayose Y. Ashikari M. Yamanouchi U. Monna L. Fuse T.. (2000). Hd1, a major photoperiod sensitivity quantitative trait locus in rice, is closely related to the *Arabidopsis* flowering time gene CONSTANS. Plant Cell 12, 2473–2484. doi: 10.1105/tpc.12.12.2473, PMID: 11148291 PMC102231

[ref171] Yin K. Qiu J.-L. (2019). Genome editing for plant disease resistance: applications and perspectives. Philos. Trans. R. Soc. B Biol. Sci. 374:20180322. doi: 10.1098/rstb.2018.0322, PMID: 30967029 PMC6367152

[ref172] Yoo S. Y. Kardailsky I. Lee J. S. Weigel D. Ahn J. H. (2004). Acceleration of flowering by overexpression of MFT (MOTHER OF FT AND TFL1). Mol. Cell 17, 95–101. 15055534

[ref173] Yu Q. Cairns A. Powles S. B. (2004). Paraquat resistance in a population of Lolium rigidum. Funct. Plant Biol. 31, 247–254. doi: 10.1071/FP03234, PMID: 32688896

[ref174] Yu Q. Huang S. Powles S. (2010). Direct measurement of paraquat in leaf protoplasts indicates vacuolar paraquat sequestration as a resistance mechanism in *Lolium rigidum*. Pestic. Biochem. Physiol. 98, 104–109. doi: 10.1016/j.pestbp.2010.05.007

[ref175] Yu Q. Liu S. Yu L. Xiao Y. Zhang S. Wang X.. (2021). RNA demethylation increases the yield and biomass of rice and potato plants in field trials. Nat. Biotechnol. 39, 1581–1588. doi: 10.1038/s41587-021-00982-9, PMID: 34294912

[ref176] Zeng Y. Wen J. Zhao W. Wang Q. Huang W. (2020). Rational improvement of rice yield and cold tolerance by editing the three genes OsPIN5b, GS3, and OsMYB30 with the CRISPR–Cas9 system. Front. Plant Sci. 10:1663. doi: 10.3389/fpls.2019.01663, PMID: 31993066 PMC6964726

[ref177] Zhang A. Liu Y. Wang F. Li T. Chen Z. Kong D.. (2019). Enhanced rice salinity tolerance via CRISPR/Cas9-targeted mutagenesis of the OsRR22 gene. Mol. Breed. 39:47. doi: 10.1007/s11032-019-0954-y, PMID: 32803201 PMC7413041

[ref178] Zhang Y. Massel K. Godwin I. D. Gao C. (2018). Applications and potential of genome editing in crop improvement. Genome Biol. 19:210. doi: 10.1186/s13059-018-1586-y, PMID: 30501614 PMC6267055

[ref179] Zhang T. Mudgett M. Rambabu R. Abramson B. Dai X. Michael T. P.. (2021). Selective inheritance of target genes from only one parent of sexually reproduced F1 progeny in *Arabidopsis*. Nat. Commun. 12:3854. doi: 10.1038/s41467-021-24195-5, PMID: 34158505 PMC8219824

